# Ketogenic Diet as a potential treatment for traumatic brain injury in mice

**DOI:** 10.1038/s41598-021-02849-0

**Published:** 2021-12-07

**Authors:** Meirav Har-Even, Vardit Rubovitch, Whitney A. Ratliff, Bar Richmond-Hacham, Bruce A. Citron, Chaim G. Pick

**Affiliations:** 1 Department of Anatomy and Anthropology, Sackler Faculty of Medicine, Tel Aviv University, 6997801 Tel Aviv, Israel; 2 Sylvan Adams Sports Institute, Tel Aviv University, 6997801 Tel Aviv, Israel; 3 Laboratory of Molecular Biology, Bay Pines VA Healthcare System, Research and Development, 10000 Bay Pines Blvd., 151, Bldg. 22, Rm. 123, Bay Pines, FL 33744-4125 USA; 4 Laboratory of Molecular Biology, Research and Development (Mailstop 15), VA New Jersey Health Care System, Building 16, Rm. 16-176, 385 Tremont Ave, East Orange, NJ 07018 USA; 5 Department of Pharmacology, Physiology and Neuroscience, Rutgers-New Jersey Medical School, Newark, NJ 07103 USA; 6 Sagol School of Neuroscience, Tel Aviv University, 6997801 Tel Aviv, Israel; 7 Dr. Miriam and Sheldon G. Adelson Chair and Center for the Biology of Addictive Diseases, Tel Aviv University, 6997801 Tel Aviv, Israel

**Keywords:** Neuroscience, Neurology

## Abstract

Traumatic brain injury (TBI) is a brain dysfunction without present treatment. Previous studies have shown that animals fed ketogenic diet (KD) perform better in learning tasks than those fed standard diet (SD) following brain injury. The goal of this study was to examine whether KD is a neuroprotective in TBI mouse model. We utilized a closed head injury model to induce TBI in mice, followed by up to 30 days of KD/SD. Elevated levels of ketone bodies were confirmed in the blood following KD. Cognitive and behavioral performance was assessed post injury and molecular and cellular changes were assessed within the temporal cortex and hippocampus. Y-maze and Novel Object Recognition tasks indicated that mTBI mice maintained on KD displayed better cognitive abilities than mTBI mice maintained on SD. Mice maintained on SD post-injury demonstrated SIRT1 reduction when compared with uninjured and KD groups. In addition, KD management attenuated mTBI-induced astrocyte reactivity in the dentate gyrus and decreased degeneration of neurons in the dentate gyrus and in the cortex. These results support accumulating evidence that KD may be an effective approach to increase the brain’s resistance to damage and suggest a potential new therapeutic strategy for treating TBI.

## Introduction

Traumatic Brain Injury (TBI) is a brain dysfunction that occurs as the result of an external force's impact on the brain (external impact, penetration, or rapid head movement). The most common causes of TBI's are military injuries, road accidents, falls, assaults, and sports injuries^1^. Approximately 2.8 million people in the United States alone receive medical care for TBI each year, amounting to an annual cost above $76 billion, including medical, unemployment, and additional costs^1,2^. Sequelae of TBI include physical, cognitive, behavioral, emotional, and social problems^3,4^. TBI emanates different pathophysiological changes that progress in two phases. The first, primary brain injury is a direct result of the external force applied to the brain; this phase consists of tissue alteration and damage proximal to the injury and interruption of axons and small vessels, which causes immediate necrotic neuronal cell death^5^. The secondary process initiated during the primary phase includes neuroinflammation, glutamate excitotoxicity and oxidative stress, which leads to neuronal apoptotic cell death^5,6^. Mild TBI (mTBI), which accounts for over 80–90% of all TBI cases, is difficult to diagnose because routine tests, including imaging, fail to show changes in brain structure^7,8^. Despite this, mTBI patients frequently suffer short and long-lasting cognitive, behavioral, and emotional impairments^9^. Such impairments include, among others, memory and concentration deficits, poor executive functions, depression, and anxiety-related disorders^10^.

The ketogenic diet (KD) is a high-fat, low carbohydrate diet, originally designed to stimulate the beneficial biochemical changes found in the fasting state, and has been used in patients with difficult-to-treat epilepsy since 1921^11^. KD is one method for inducing ketosis, a metabolic state in which the body uses ketone bodies as energy supply instead of glucose, the primary substrate for energy metabolism in the body and brain. Rodents receiving KD have shown a long-lasting affect of rapid increase in ketone body levels that lasted up to eight weeks into their diet^12^. Recent studies have begun to explore ketogenic therapies in other neurological and psychiatric disorders, including Parkinson's disease^13^ and Alzheimer's disease^14,15^. An increasing number of murine studies suggest that KD induces anti-inflammatory effects^16^. Studies have reported that KD, and in particular, the ketone metabolite beta-hydroxybutyrate suppresses activation of the NLRP3 inflammation in response to several structurally unrelated NLRP3 activators^16^ and improves the brain’s ischemic tolerance^17^. KD management has also been shown to reduce activated microglial expression^18^. While mechanism of KD in neuroprotection is unknown, past reports found that KD increases glutathione level^19^ and Uncoupling protein (UCP)^20^ in cells after brain injuries, decreasing Reactive Oxygen Species (ROS).

A possible mechanism by which KD could be inducing neuroprotective effects is through the alteration of SIRT1 expression. SIRT1 has a role in developing the hippocampus by activating Akt and inhibiting GSK3 and is involved in various physiological processes such as oxidative stress response, genetic silencing, genome stability, and cell life extension^21,22^. The Sirtuin family of proteins is also active in the hypothalamus, where it plays a role in regulating circadian rhythm, endocrine pathways, and appetite^23–26^. Recently, several studies have shown that SIRT1 plays a significant role in induced neuroprotection following caloric restriction (CR)^27,28^. CR has also been linked to reduced tau phosphorylation and maintenance of hippocampal neurons^29^, and related Tau pathology is known to be implicated in different types of TBI^30,31^. While CR has been shown to increase ketone bodies in the blood, KD does this at a higher level^32^. This is important to note as high fat diet and increased circulating ketone bodies have been shown to activate SIRT1^33^. Thus, the effects of SIRT1 following KD, as in our model, may be more pronounced than in previous models utilizing CR. Our present study utilizing adult male ICR mice coincides with past rodent studies addressing SIRT1^34–36^.

In the present study, we utilize a closed head weight drop model of murine mTBI to test the cognitive, cellular, and molecular effects of up to 30 days of KD management following injury. We report that KD initiated after mTBI ameliorated the cognitive deficits in spatial and visual memory, as well as cellular changes in neurons and glial cells induced by the injury. Our model also shows that KD sustained the levels of SIRT1 expression which were decreased with injury.

## Results

### KD increased the level of ketone bodies in blood

**Figure 1:**
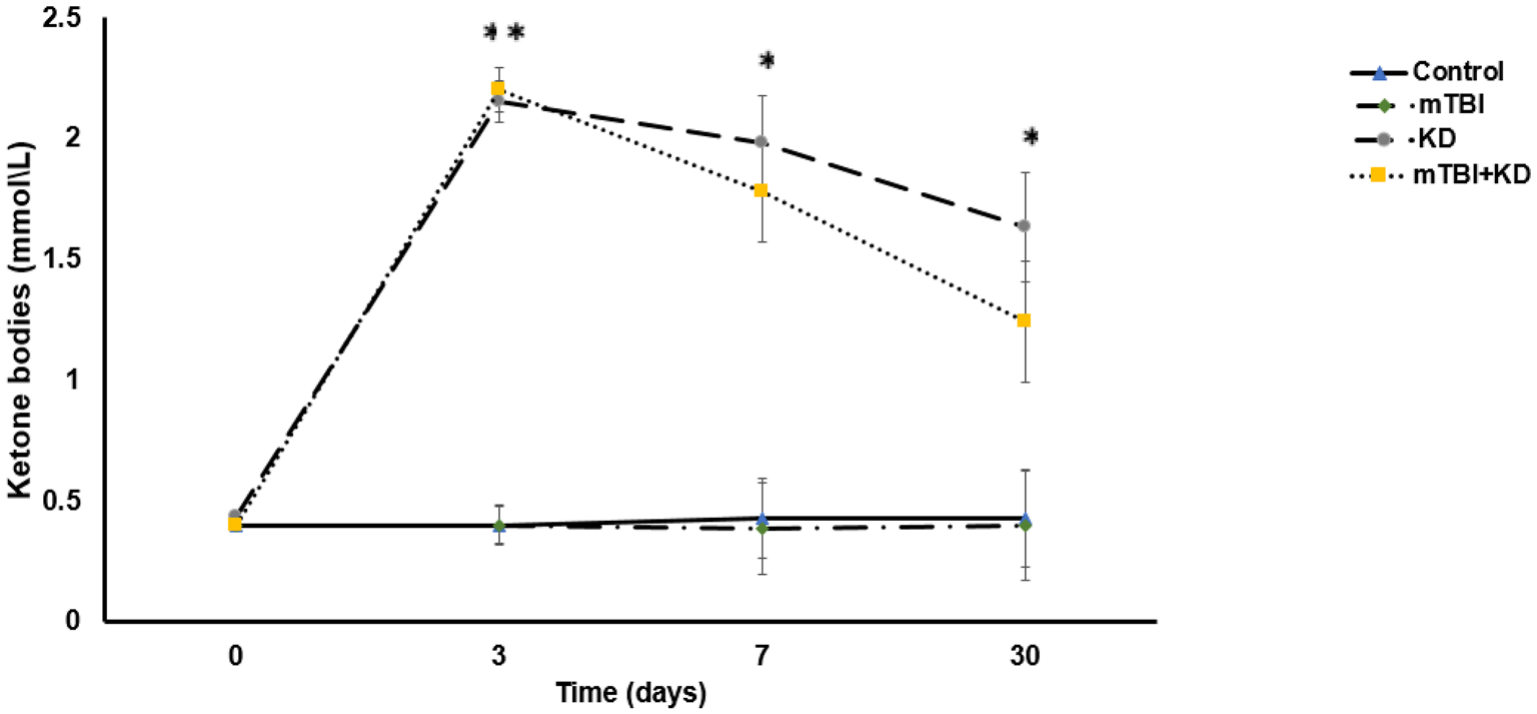
Levels of blood ketone bodies of control (n = 8), mTBI (n = 6), KD (n = 6), and mTBI + KD (n = 5) mice. Mice that received KD (with or without mTBI) demonstrated a prominent increase in ketone bodies compared to mice fed SD at 3, 7, and 30 days after the diet was initiated.

One-way repeated ANOVA revealed a significant main effect of time [F (3,63) = 28.31, p = 0.000, η^2^ = 0.57] and group [F (3,21) = 49.24, p = 0.000, η^2^ = 0.88] as well a time by group interaction [F (9,63) = 9.81, p = 0.000, η^2^ = 0.58]. Consecutive simple effects analysis with Sidak test indicated that the level of ketone bodies in the blood of KD mice was significantly higher than the level in mTBI and control mice at the 3-day time-point (both p = 0.000), 7-day time-point (both p = 0.000), and 30-day time-point (both p = 0.001). Similarly, the blood ketone levels in the mTBI + KD group were persistently higher than that of the mTBI and control groups at the 3-day time-point (both p = 0.000), 7-day time-point (both p = 0.001), and 30-day time-point (p = 0.022 and p = 0.018, respectively). See Fig. 1.

### mTBI exposure and KD management does not affect anxiety

The EPM test was applied to assess anxiety-like behavior. Two-way ANOVA demonstrated no significant main effect of group [F (3, 76) = 1.61, p = 0.194, η^2^ = 0.06] or time-post injury [F (1, 76) = 3.33, p = 0.072, η^2^ = 0.04], and no interaction between the two factors [F (3, 76) = 0.55, p = 0.649, η^2^ = 0.02].

**Figure 2:**
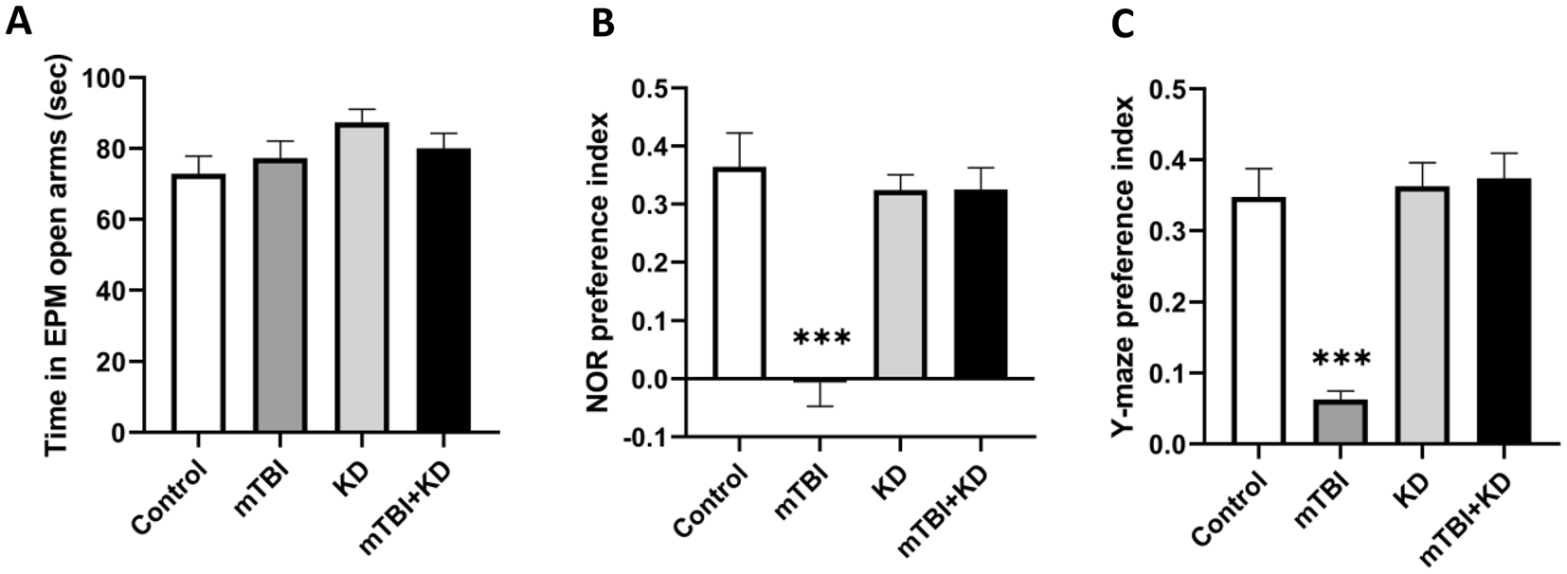
Behavioral tests scores. (**A**) EPM test—the time spent in open arms did not differ between mice in the control (n = 19), KD (n = 23), mTBI (n = 18), and mTBI + KD (n = 24) groups, indicating that mTBI did not affect anxiety-like behavior. (**B**) NOR test—differences in visual recognition memory performance between mice in the control (n = 19), KD (n = 23), mTBI (n = 18), and mTBI + KD (n = 24) groups. (**C**) Y-maze test- differences in spatial memory performance between mice in the control (n = 20), KD (n = 24), mTBI (n = 19), and mTBI + KD (n = 26) groups.

The mice's anxiety-like behavior was not affected either by the injury or diet management. See Fig. 2A.

### KD ameliorates cognitive deficits

The NOR test was used to assess visual recognition memory. Two-way ANOVA revealed a significant main effect of group [F (3, 76) = 17.11, p = 0.000, η^2^ = 0.40]. Gabriel’s post‐hoc analysis demonstrated that the mTBI group performed significantly worse as compared to the controls, KD, and mTBI + KD groups (all p = 0.000). There was no main effect of time-post injury [F (1, 76) = 0.60, p = 0.442, η^2^ = 0.01] or group by time-post injury interaction [F (3, 76) = 2.30, p = 0.084, η^2^ = 0.08]. See Fig. 2B.

The Y-maze test was used to assess spatial memory. Two-way ANOVA showed a significant main effect of group [F (3, 81) = 19.49, p = 0.000, η^2^ = 0.42]. Gabriel’s post‐hoc analysis showed that mice in the mTBI group performed significantly worse than mice in the control, KD, and mTBI + KD groups (all p = 0.000). There was no main effect of time-post injury [F (1, 81) = 0.46 p = 0.498, η^2^ = 0.01] nor a group by time-post injury interaction [F (3, 81) = 0.75, p = 0.526, η^2^ = 0.03]. See Fig. 2C.

### KD prevents the reduction of SIRT1 expression following mTBI

**Figure 3:**
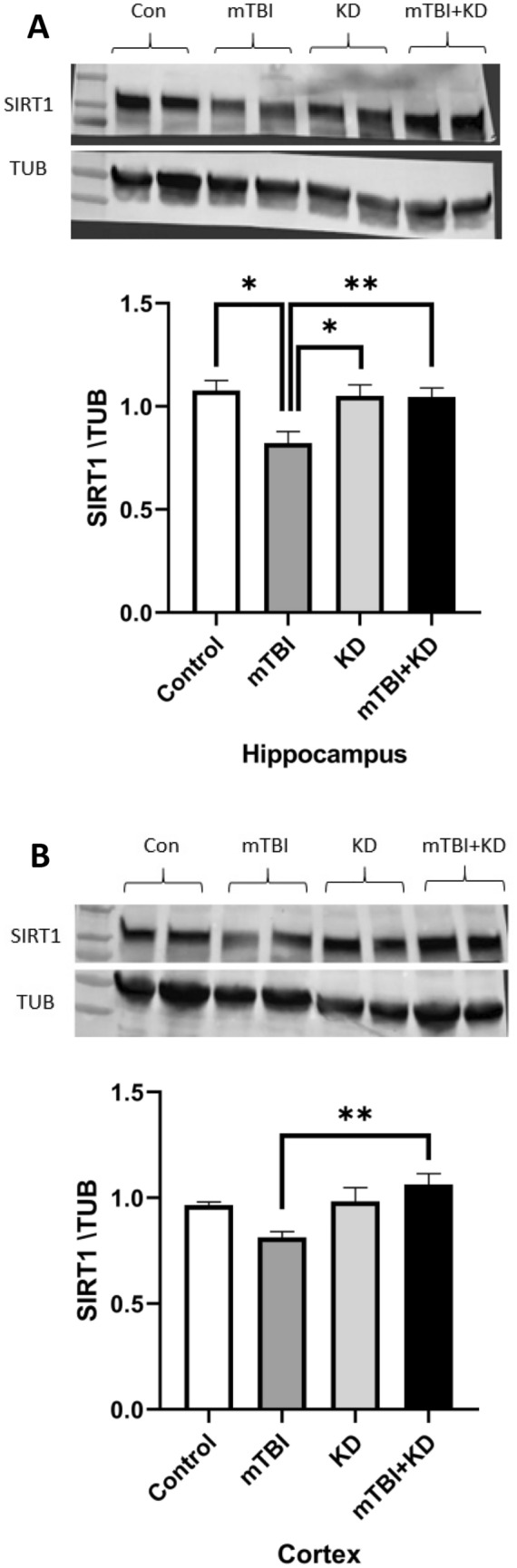
Changes in SIRT1 expression after mTBI in the cortex and hippocampus of control (n = 7), KD (n = 5), mTBI (n = 6), and mTBI + KD (n = 7) mice. (**A**) The levels of SIRT1 were significantly reduced in the hippocampus of mTBI mice compared with all groups. (**B**) The levels of SIRT1 were significantly reduced in the cortex of mTBI mice compared with all mTBI + KD group. The membrane was cropped immediately after the transfer stage due to the usage of two different antibodies on the same blot membrane, SIRT1 band size was at 110 kDa and α-tubulin band size was at 55 kDa, full-length blots/gels are presented in Supplementary Figs. 1–4.

SIRT1 expression within the hippocampus- one-way ANOVA revealed significant between-group differences in SIRT1 expression [F (3,21) = 5.45, p = 0.006, η^2^ = 0.44]. Gabriel’s post‐hoc analysis showed that the mTBI group had significantly lower SIRT1 levels than the control (p = 0.009), KD (p = 0.038) and mTBI + KD (p = 0.025) groups. See Fig. 3A.

SIRT1 expression within the cortex-one-way ANOVA revealed significant between-group differences in SIRT1 expression [F (3,21) = 6.26, p = 0.003, η^2^ = 0.47]. Gabriel’s post‐hoc analysis showed that the mTBI group had significantly lower SIRT1 levels than the mTBI + KD group (p = 0.002). In addition, the mTBI group showed a marginally significant trend toward lower SIRT1 levels than the than the control (p = 0.090) and KD (p = 0.077) groups. See Fig. 3B.

### Ketogenic Diet prevents mTBI-induced neuronal loss

**Figure 4:**
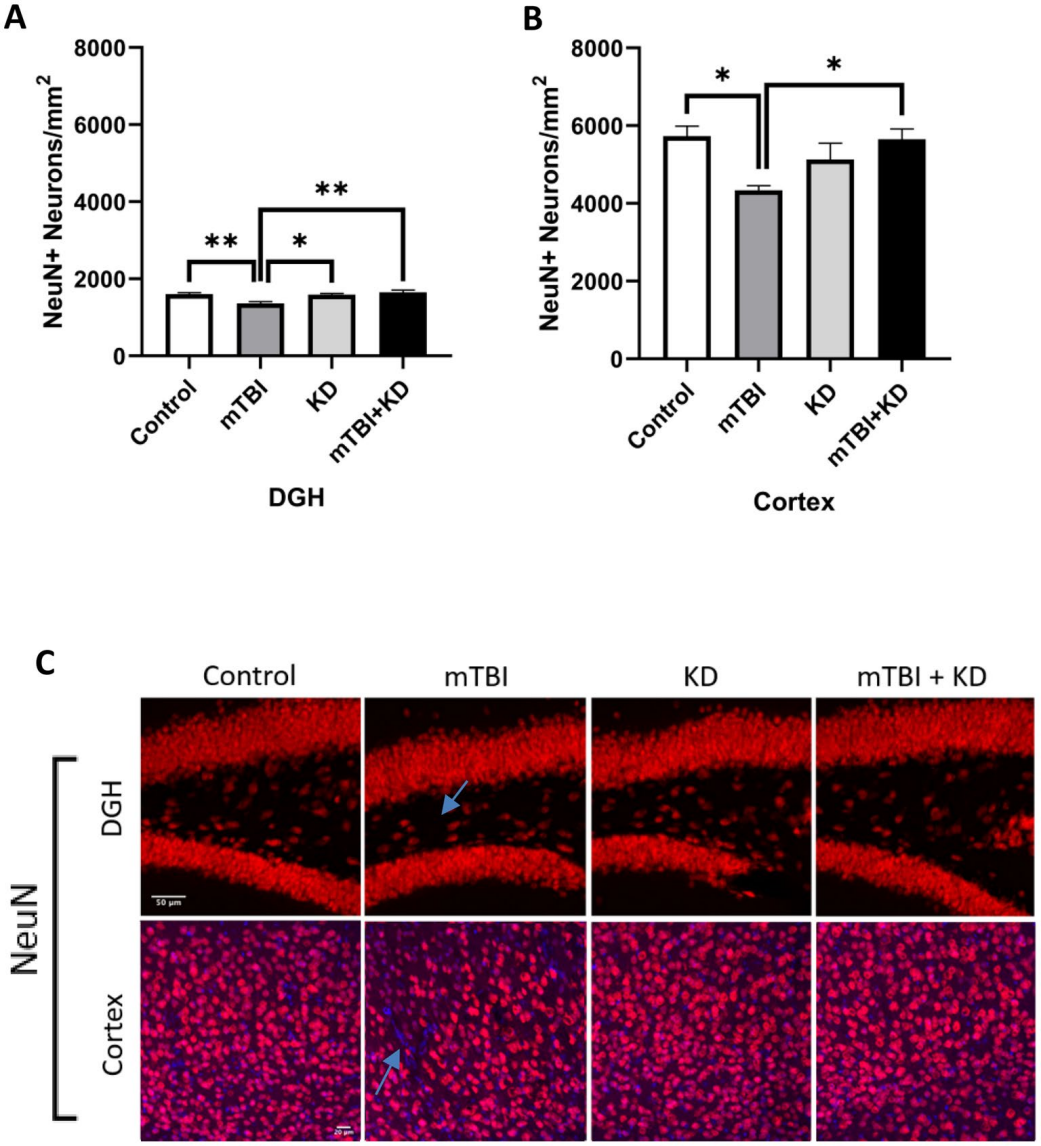
NeuN^+^ changes in the cortex and hippocampus of control (n = 5), KD (n = 5), mTBI (n = 5), and mTBI + KD (n = 5) mice. (**A**,**B**) mTBI induction led to a significant decrease in the density of NeuN^+^ neurons compared to sham tissues in all regions tested. With KD, there were significantly greater numbers of NeuN^+^ neurons than in the untreated mTBI mice, suggesting an increase in neuronal survival following the injury. (**C**) Representative images of immunohistochemical staining in the DGH and the temporal cortex are presented. NeuN positive cells are shown in red, and nuclei in blue; the scale bars are 50 μm in the DGH and 20 μm in the temporal cortex.

The number of NeuN + neurons in the cortex and dentate gyrus-one-way ANOVA revealed significant between-group differences in the number of NeuN + neurons both within the cortex [F (3, 16) = 5.06, p = 0.012, η^2^ = 0.49] and the dentate gyrus [F (3, 16) = 8.51, p = 0.001, η^2^ = 0.61]. Gabriel’s post‐hoc analysis demonstrated that the total number of NeuN + neurons within the cortex was significantly lower in the mTBI group than in the control (p = 0.018) and mTBI + KD groups (p = 0.028). The total number of NeuN + neurons within the dentate gyrus was significantly lower in the mTBI group than in the control (p = 0.008), KD (p = 0.012), and mTBI + KD (p = 0.002) groups. See Fig. 4(A–C).

### Ketogenic Diet mitigates TBI-induced neuroinflammation by reducing reactive astrocytes

**Figure 5:**
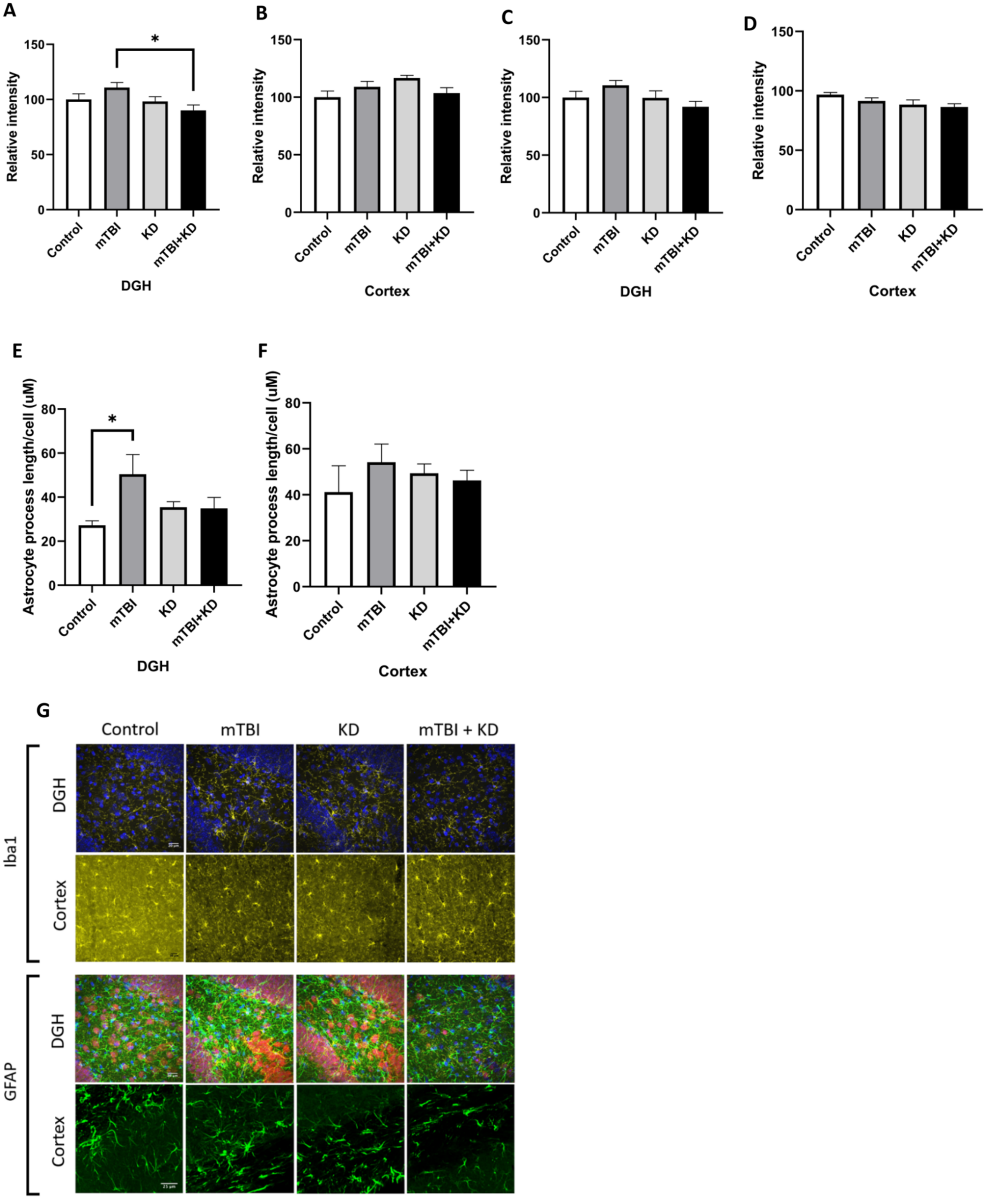
Microglia and astrocyte changes in the cortex and hippocampus of control (n = 5), KD (n = 5), mTBI (n = 5), and mTBI + KD (n = 5) mice. KD reduces the mTBI-induced elevation in active astrocyte expression in the DGH only and not in activated microglia expression at 30 days post-injury. Graphs present quantification of the total surface labeled with (**A**) GFAP in the DGH (**B**) GFAP in the temporal cortex (**C**) Iba-1 in the DGH (**D**) Iba-1 in the temporal cortex. In addition, (**E**,**F**) Astrocyte morphology in the DG Hilus region displayed approximately twice the ramification as the control samples. (**G**) Representative images of immunohistochemical staining in the DGH and the temporal cortex are presented. GFAP positive cells are shown in green, Iba-1 positive cells are shown in yellow, and NeuN+ cells in red. The scale bars are 20–25 μm.

GFAP Intensity in the cortex and dentate gyrus- one-way ANOVA revealed significant between-group differences in GFAP intensity in the dentate gyrus [F (3, 16) = 3.14, p = 0.055, η^2^ = 0.37], but not in the cortex [F (3, 16) = 2.60, p = 088, η^2^ = 0.33]. Gabriel’s post‐hoc analysis demonstrated that the GFAP intensity in the dentate gyrus was higher in the mTBI group than in the mTBI + KD group (p = 0.043). See Fig. 5A,B.

GFAP Morphology in the cortex and dentate gyrus-one-way ANOVA revealed significant between-group differences in GFAP Morphology in the dentate gyrus DGH- [F (3, 16) = 3.28, p = 0.048, η^2^ = 0.38], but not in the cortex [F (3, 16) = 0.52, p = 0.674, η^2^ = 0.09]. Gabriel’s post‐hoc analysis revealed marked alterations in astrocyte morphology in the dentate gyrus of mTBI mice versus controls (p = 0.042). See Fig. 5(C,D).

Activated microglia expression in the cortex and dentate gyrus-one-way ANOVA revealed no significant differences between groups in the number of activated microglia within the cortex [F (3, 16) = 2.37, p = 0.108, η^2^ = 0.31] or dentate gyrus [F (3, 16) = 2.15, p = 0.134, η^2^ = 0.29]. See Fig. 5E,F.

Images of immunohistochemical staining in the DGH and the temporal cortex with GFAP positive cells shown in green, Iba-1 positive cells shown in yellow, and NeuN + cells in red. See Fig. 5G.

## Discussion

TBI is a leading cause of death and long-term disability in the developed world, with more than 10 million people suffering worldwide every year^37^. The majority of these TBIs (80–95%) are mild in nature^7,8^. TBI symptoms can occasionally resolve within the first year after injury, but up to 70–90% of patients continue to manifest prolonged and often permanent neurocognitive dysfunction. In light of the growing reports suggesting beneficial effects of KD in many neurological disorders^13–15^, the primary goal of this study was to assess the benefits of KD in mTBI. Our results suggest that KD may be a vital treatment modality, mitigating TBI-induced cognitive impairments, neuronal loss, and neuroinflammation in the closed-head mTBI mouse model.

We delivered a mild TBI to mice followed by up to 30 days of KD or SD. We were able to confirm that mice who received KD (with or without mTBI) demonstrated a prominent increase in ketone bodies compared to mice fed SD at 3, 7, and 30 days after the diet. Previous studies have demonstrated that mice challenged with mTBI show cognitive impairments in visual and spatial memory^10,38^. Our previous published studies as well as the present study, ruled out the possible involvement of anxiety (as assessed by EPM) in the cognitive performance of the injured mice. This study's results replicated the previous findings regarding both visual and spatial memory while demonstrating that KD significantly ameliorated these cognitive deficits. These results support previous studies on the benefits of KD on brain injury in the short-term (7 days); rats who were fed KD exhibited evidence of neuroprotection after head trauma in the cortex and hippocampus^39^ and improvement in motor performance in Beam balance and Beam walk tests^18^, KD administration has also been shown to reduce brain edema and cellular apoptosis 72 h after TBI^40^ and has demonstrated anti-tumor effects within the brain in rodent models^41^.

To better understand the molecular effects of TBI and KD, we evaluated SIRT1 levels in two brain regions, cortex and hippocampus, which play crucial roles in memory formation. TBI reduced the levels of SIRT1 in cortex and in the hippocampus 30 days post-injury which was ameliorated by KD, suggesting a potential mechanism contributing to cognitive impairment and improvement in cognitive symptoms when given KD^42^. In contrast, at 7 days post-injury SIRT1 levels were not reduced in the cortex and hippocampus. This supports past findings showing an elevation in SIRT1 levels 1 day prior to ischemia that gradually decreased over a period of 7 days^36^. Our study's results align with prior studies of intermittent fasting and caloric restriction for 30 days post-injury^28^ and with research involving KD and SIRT1 in health-enhancing, aging, longevity, and neurodegeneration in animal models^43,44^.

We have previously reported that our mTBI model decreases the neuronal survival 3 weeks post injury^45^. Similarly, in the present study, we have shown a significant increase in neuronal cell death that persists 30 days following injury in both cortex and hippocampus. KD prevented this neuronal cell death. These results are consistent with an in vitro study that found the ketone body beta-hydroxybutyrate reduced axonal degeneration in diffuse axonal injury^46^. Additionally, we saw a marked increase in reactive astrocytes in hippocampus 30 days following injury, which was ameliorated by KD. We have previously reported that our mTBI model induces fundamental neuroinflammatory changes, including elevations in astrocyte reactivity, pro-inflammatory cytokine TNF-α levels, and expression of genes involved in inflammatory processes in several regions of the brain^44,47–50^. We can conclude that KD prevented the injury-induced neuro-inflammation, which is in agreement with previous studies that reported KD inhibited NLRP3 inflammasome activation, thus exerting neuroprotective effects^17^.

Our closed head injury mouse model of mild TBI was able to recapitulate the cognitive deficits observed in human TBI patients^51,52^ and showed that ketogenic diet beginning following injury could protect against injury-induced memory loss. At the cellular level, we have demonstrated that mTBI induced neuronal cell death, astrocyte and microglial activation (neuro-inflammation), which were prevented in injured mice treated with KD. Our analysis of changes in SIRT1 suggests a viable molecular mechanism by which KD may be improving cognitive outcomes. Our results support accumulating evidence that KD may represent an important non-pharmaceutical treatment against the long-term molecular and cellular impacts of mTBI, which may ultimately improve cognitive symptoms and quality of life for TBI patients.

## Methods

### Experimental procedures

**Figure 6:**
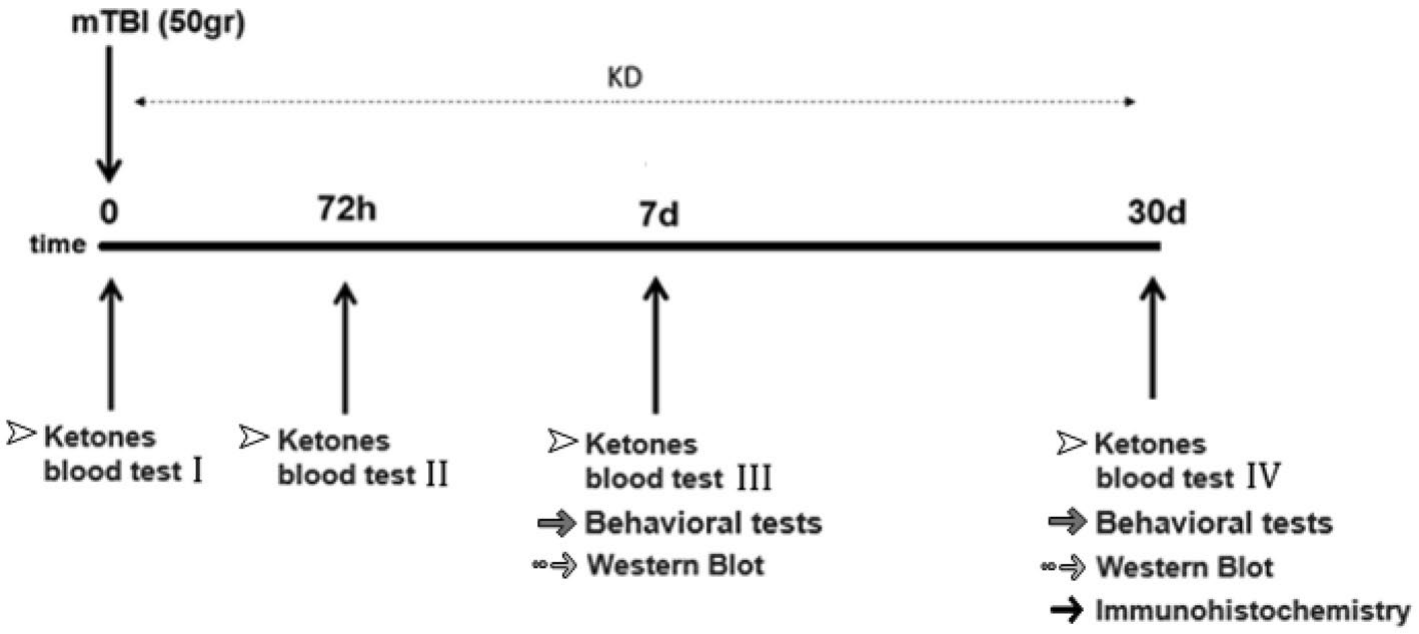
Study timeline. Animals were exposed to mTBI or sham and checked for ketone bodies in the blood at days 0, 3, 7 and 30. Mice were then fed KD/SD for 3, 7, or 30 days. Behavioral tests to assess behavior and cognitive abilities were carried out at 7 and 30 days following mTBI in separate cohorts. Western blot analysis to assess changes in SIRT1 levels following mTBI and diets management was performed at 7 and 30 days post-mTBI. Immunohistochemical staining to evaluate neurodegeneration and neuroinflammation was performed at 30 days following the mTBI challenge.

Mice were subjected to mTBI, and a ketogenic diet (KD) was initiated immediately for the following three timelines: 3 days, 7 days, and 30 days. Ketone bodies in the blood were measured 0, 3, 7, and 30 days post-injury. Behavioral tests were performed at 7 or 30 days following mTBI in separate groups of animals. Western blot analysis was carried out on brain tissue collected from 7 and 30 days post mTBI. Immunohistochemical staining assessments were performed on brains that were collected 30 days post-injury. The timeline of the experimental procedures following exposure to mTBI is shown in Fig. 6.

### Animals

The Sackler Commission on Animal Experimentation ethical committee approved the study and animal protocol 01-19-058 according to the Guidelines for Animal Experimentation of the National Institutes of Health (DHEW publication 23-85, revised, 1995). The study complies with ARRIVE guidelines.

Male ICR mice, aged 6–7 weeks, 30–40 g body weight, were acquired from Envigo RMS Israel. Mice were housed at 4–5 per home cage under a constant 12 h light/dark cycle, at room temperature (22 ± 2 °C) in a standard plastic cage (32 × 21.5 × 12 cm^3^). Water and food was provided ad libitum, and the cages bedding, sterile sawdust, was replaced once per week. All mice were acclimatized to the facility for three days following transport and then moved into the experimental testing room for three days prior to experimentation. Animals were utilized only once throughout the study in either behavioral, biochemistry, or immunocytochemistry tests. Each animal in a given group was tested only once in order to avoid the possible confounding effect of behavioral testing. The number of animals evaluated in each assessment group and the measurement times performed was based on analysis of variance in our previous studies.

### Mouse closed-head mild traumatic brain injury

Mild Traumatic Brain Injury (mTBI) was induced according to the closed-head weight-drop model, as employed in our previous studies^53,54^. The device consisted of an aluminum tube (80 cm in length and 13 mm in diameter). In the pre-injury stage, mice were anesthetized by inhalation of isoflurane and placed under the device on a sponge, the sponge supported the head of the mouse allowing some anterior–posterior motion, but no rotational head movement at the moment of impact. A metal weight (50 g) was dropped from the top of the tube to strike the head on the right temporal side between the ear and corner of the eye. Sham mice received anesthesia and were placed on the sponge for an equivalent length of time, but no weight was dropped. This model was chosen because it simulates traumatic head injuries such as road accidents or falls, as it imposes a diffuse and non-specific injury. Our model, while subject to a certain amount of variability due to the natural anatomic variation, still maintains a high degree of TBI injury similarity among all mice as seen in previous neuronal loss models^53–56^.

### Ketogenic Diet

The macronutrient composition of the Ketogenic Diet (TD.96355; Harlan Teklad Laboratories, Madison, WI) was 9.2% protein, 0.3% carbohydrate, and 90.5% fat (% kcal) (Table 1)^57–59^. The corresponding components of the standard diet (SD) (TD.00606; Harlan Teklad Laboratories, Madison, WI) were 10.1% protein, 77.4% carbohydrate, and 12.5% fat (% kcal)^57^. The diets and water were provided ad libitum for 3, 7, and 30 days after the injury. Both diets were stored at 4 °C, KD, which had a solid, butter-like texture, was changed every day, and SD pellets were changed twice per week.

**Table 1:** Macronutrient information of each diet.

% Kcal from	Standard diet	Ketogenic diet
Fat	12.5	90.5
Carbohydrate	77.4	0.3
Protein	10.1	9.2

### Measurement of Ketone Bodies

Ketone bodies in the blood were measured using the Precision Xtra Blood Glucose and Ketone Monitoring System (Abbott, Columbus, OH)^60^. Mice were anesthetized with isoflurane and tails were cut approximately 0.2 mm from the end, a drop of blood was squeezed directly into a testing strip attached to the measuring instrument. Group sizes were as follows: control (n = 8), mTBI (n = 6), KD (n = 6), and mTBI + KD (n = 5).

### Elevated plus maze

The elevated plus maze (EPM) was used to evaluate anxiety-like behavior^61^. This assessment relies on the natural anxiety-like behavior exhibited by rodents when placed in brightly lit, open environments. The maze is plus-shaped (+), with arms extending from the center at 90° angles from each other. Opposite arms in the plus formation are identical, with two open arms and two closed (walled) arms measuring 30 × 5 × 1 cm each. During testing, each mouse was placed at the maze's center, facing one of the open arms, and was allowed to explore the maze for 5 min. The amount of time the mice spent in the open arms and the number of entries to the closed and open arms was counted. A longer duration of time spent within the open arms has been associated with lower anxiety levels^61,62^. Group sizes were as follows: control (n = 19), mTBI (n = 18), KD (n = 23), and mTBI + KD (n = 24).

### Novel object recognition

The novel object recognition (NOR) task assesses recognition and visual memory^63^. This paradigm relies on rodents' natural tendency to investigate novel objects within their environment rather than known ones. NOR evaluates whether a mouse is able to discriminate between a familiar and a novel object. The testing arena is a square surface (60 cm × 60 cm) with high walls (20 cm). The test consists of three 5 min sessions, separated by 24 h. On the first day, mice were individually put in the empty arena for habituation for 5 min. On the second day, the mice were exposed to 2 identical objects within the arena for 5 min. On the third (experimental) day, one of the familiar objects was replaced with a novel object, and mice were allowed to explore the arena again for 5 min, during which time spent near familiar and novel object was measured. The arena was cleaned with 70% ethanol between subjects. An Aggelton index was calculated as follows: (time near new novel object − time near familiar object)/(time near new novel object + time near familiar object)^64^. A higher Aggelton index indicates advancement in recognition memory. Animals near the objects less than 10% of the total test time (i.e., less than 30 s next to the two objects together) were excluded from statistical calculations, as exploration under this amount of time does not allow estimation of subjects' visual memory. Group sizes were as follows: control (n = 19), mTBI (n = 18), KD (n = 23), and mTBI + KD (n = 24).

### Y-maze

The Y-maze paradigm was used to evaluate spontaneous exploration, responsiveness to novel environments, and spatial memory function, as previously described^65^. This test relies on the preference of rodents to explore new environments rather than familiar ones. The Y-maze consists of a three-armed black plexiglass maze with arms separated by 120°. Each arm was identical (8 × 30 × 15 cm); however, different spatial cues were placed in each arm (i.e., a triangle, a square, or a circle). In the first session of the test, the mouse was put in the arena's start arm (chosen randomly) and allowed to explore another arm while the third arm was blocked for 5 min. The mouse was then returned to its home cage for 2 min. In the second session, all arms were open for exploration for 2 min and time spent in each arm was recorded. The arena was cleaned with 70% ethanol between subjects. The time the mouse spent in the familiar arm and the new arm was measured. An Aggelton index was calculated as follows: (time in the new arm − time in the familiar arm)/(time in new arm + time in the familiar arm)^64^. A higher Aggelton index indicates improved spatial memory. Group sizes were as follows: control (n = 20), mTBI (n = 19), KD (n = 24), and mTBI + KD (n = 26).

### Immunohistochemistry

Immunohistochemistry studies were performed hippocampal (dentate gyrus) and temporal cortex tissue sections obtained from animals euthanized on day 30 post-injury. Mice were anesthetized with ketamine (100 mg/kg) and xylazine (10 mg/kg) and underwent transcardial perfusion with 10 ml phosphate-buffered saline (PBS) followed by 20 ml of 4% paraformaldehyde (PFA) in 0.1 M phosphate buffer, pH 7.4. Brains were removed, fixed overnight in 4% PFA, and then placed in 1% PFA. Neuroscience Associates (Knoxville, TN) oriented the brains into a multiblock, collected 35 μm sections sequentially through the brains, and performed the floating section staining and mounting (antibodies detailed in Table 2). Microscopy was performed with a Fluoview 3000 laser scanning confocal microscope (Olympus, Waltham, MA). Target locations were determined on a stitched map with only Hoechst 33342 staining captured. For all analyses, regions of interest were selected on the map depicting only the nuclear staining and blinded with respect to groupings. These regions were then collected by multi area time lapse in sequence with the Fluoview 3000 software without intervention. Images, centered on coronal sections at approximately − 2.9 mm from Bregma, were collected as Z stacks and maximum Z projections with constant illumination (405, 488, 561, and 640 nm diode lasers) and detection parameters. Automated analysis for morphology, intensities, and numbers of cells was conducted using cellSens (Olympus, Waltham, MA, USA) and ImageJ^66,67^.

**Table 2:** Immunohistochemistry reagents.

Target/fluorochrome	Primary/secondary	Probe	Manufacturer	Catalog	Dilution
Nuclei		Hoechst 33342	ThermoFisher		
Astrocytes	Primary	Chicken anti-GFAP	Encor	CPCA-GFA	1:1500
Alexa488	Secondary	Donkey anti-chicken	Jackson	703-545-155	1:500
Microglia	Primary	Goat anti-Iba1	ThermoFisher	PA5-18039	1:1500
Alexa555	Secondary	Donkey anti-goat	ThermoFisher	A21432	1:500
NeuN + Neurons	Primary	Rabbit anti-NeuN	Abcam	Ab104225	1:5000
Alexa647	Secondary	Donkey anti-rabbit	ThermoFisher	A31573	1:500

Changes in the architecture of astrocytes were determined by batch processing of all images collected with the aid of ImageJ. All of the following steps were iteratively executed for each micrograph in a single macro. Each image was analyzed for the channel containing GFAP staining. Background was subtracted with ImageJ’s built-in rolling ball process, automated thresholding was accomplished with the built in RenyiEntropy algorithm, and noise was removed using the ImageJ despeckle routine. The image was then skeletonized with the ImageJ plugin andfeatures that were too small to be relevant were removed by the particle remover plugin. The analyze skeleton plugin generated the process lengths per cell. These routines have previously been shown to identify reactive morphology^66,67^.

Frames included in figures are correct concerning orientation, i.e., dorsal at the top, left, and right. Confocal scanning was rotated 30° to optimize the framing of regions of interest. Group sizes were as follows: control (n = 5), mTBI (n = 5), KD (n = 5), and mTBI + KD (n = 5).

### Western blotting

To assess the cortical and hippocampal SIRT1 levels, brains were dissected following cervical dislocation at 7 and 30 days post-injury. The cortex and hippocampus were separated and frozen in liquid nitrogen, then stored in − 80 °C. Prior to analysis, brains were homogenized in lysis buffer (Tissue Protein Extraction Reagent, Pierce, Waltham, MA, USA) supplemented with a protease inhibitor cocktail (Halt Protease Inhibitor Cocktail, Sigma Aldrich, St. Louis, MO, USA) using a Teflon pestle homogenizer. Homogenates were centrifuged for 15 min at 4 °C 14,000 r/min, supernatant liquids were separated from the precipitates and stored at − 80 °C. Sample buffer was added to the samples and then stored at − 18 °C. Prior to analysis, samples were heated to 90 °C for 3 min and 30 µl of each sample was then loaded and run on 4–20% Mini-Protean TGX gels (Bio-Rad, Hercules, CA, USA) followed by transfer onto nitrocellulose membranes (Bio-Rad, Hercules, CA, USA) by a transfer system (Trans-Blot Turbo, Bio-Rad, Hercules, CA, USA). Afterward, blots were blocked for 1 h at room temperature, with Tris-buffered saline, containing 0.01% Tween-20 and 5% BSA or powdered milk. Membranes were then incubated overnight at 4 °C with a mouse primary anti-SIRT1 antibody (Abcam, Cambridge, UK, ab10304, 1:500) and washed with TBS. Membranes were then incubated at room temperature for 1 h with goat anti-mouse antibody (Jackson ImmunoResearch Laboratories, Inc., West Grove, PA, 115-035-003, 1:10,000). Bands were then exposed using enhanced chemiluminescence with ECL (Millipore, Billerica, MA, United States) for 1 min by Viber Fusion FX7 imaging system (Viber Lourmat, France). Densitometry analysis of the detected signal was performed using ImageJ software. Uniform loading was verified by stripping and re-probing with a mouse primary α-tubulin antibody for 30 min at room temperature (Santa Cruz Biotechnology, Dallas, TX, sc-53030, 1:10,000), then conjugated goat anti-mouse antibody (Jackson ImmunoResearch Laboratories, Inc., West Grove, PA, 115-035-003, 1:10,000). The ratio of SIRT1 and α-tubulin (TUB) determined the value of each sample. Averages of control values in each membrane were set to 1, and all other samples were calculated accordingly^28^. Figure 3A,B shows cropped blots, the membrane was cropped immediately after the transfer stage due to the usage of two different antibodies on the same blot membrane, SIRT1 band size was at 110 kDa and α-tubulin band size was at 55 kDa, enabling the process of cropping and incubating two different antibodies due to the distantness from each other without risk of damaging the membranes pristine state. Full-length blots/gels are presented in Supplementary Figs. 1–4. Group sizes were as follows: control (n = 7), mTBI (n = 6), KD (n = 5), and mTBI + KD (n = 7).

### Statistical analysis

Statistical analysis was carried out using IBM SPSS version 24.0. All values are presented as the mean ± standard error of the mean (SEM). Statistical analysis included data imputation in order to maximize power, followed by one-way ANOVA, repeated measures ANOVA, or two-way ANOVA where appropriate. Gabriel and Sidak (α = 0.05) tests were used as post hoc tests. Partial eta squared (η^2^) was calculated to show effect size. Significance was determined as a two-sided p < 0.05. Descriptive statistics for blood ketones are provided in supplementary files, Table 1. Descriptive statistics for SIRT1 expression, Immunohistochemistry, and behavioral data are provided in supplementary files, Table 2.

### Ethical approval

The Sackler Commission on Animal Experimentation approved the animal protocol 01-19-058 according to the Guidelines for Animal Experimentation of the National Institutes of Health (DHEW publication 23-85, revised, 1995).

## Supplementary Information

Supplementary Information.

## Data Availability

All data supporting this study and its findings are available within the article or from the corresponding author upon reasonable request.
