# Supplementary material for: Ketogenic Diet as a potential treatment for traumatic brain injury in mice
Source: Sci Rep. 2021 Dec 7;11:23559. doi: 10.1038/s41598-021-02849-0 (PMC8651717; doi:10.1038/s41598-021-02849-0)
Supplement: Supplementary file 1 — Supplementary Information. [file 41598_2021_2849_MOESM1_ESM.docx]

**Supplementary Information**

**Table 1.**

Descriptive statistics for blood ketones

| **Time point** |  | **Controls**  **(n = 8)** | **mTBI**  **(n = 6)** | **KD**  **(n = 6)** | **mTBI + KD**  **(n = 5)** |
| --- | --- | --- | --- | --- | --- |
|  |  |  | Mean ±  SD |  |  |
| Day 0 |  | 0.40±  0.08 | 0.40±  0.06 | 0.43±  0.05 | 0.40±  0.07 |
| Day 3 |  | 0.40±  0.05 | 0.40±  0.06 | 2.15±  0.36 | 2.20±  0.24 |
| Day 7 |  | 0.43±  0.09 | 0.38±  0.04 | 1.98±  0.85 | 1.78±  0.47 |
| Day 30 |  | 0.43±  0.09 | 0.40±  0.06 | 1.63±  1.11 | 1.24±  0.29 |

Note. M and SD represent mean and standard deviation, respectively.

**Table 2.**

Descriptive statistics for SIRT1 expression, Immunohistochemistry, and behavioral data.

| **Measure** |  | **Controls** | **mTBI** | **KD** | **mTBI + KD** |
| --- | --- | --- | --- | --- | --- |
|  |  |  | Mean ±  SD |  |  |
| SIRT1  cortex |  | 0.97±  0.04 | 0.81±  0.07 | 0.98±  0.15 | 1.06±  0.14 |
| SIRT 1  hippocampus |  | 1.08±  0.13 | 0.82±  0.14 | 1.05±  0.12 | 1.05±  0.12 |
| NeuN  cortex |  | 5730.20±  574.46 | 4337.00±  266.55 | 5131.85±  932.68 | 5651.15±  593.84 |
| NeuN  dentate gyrus |  | 1604.20±  90.27 | 1366.40±  95.14 | 1593.20±  64.20 | 1651.41±  129.84 |
| Microglia  cortex |  | 96.83±  4.06 | 91.52±  5.94 | 88.43±  9.01 | 86.40±  6.36 |
| Microglia  dentate gyrus |  | 100.00±  12.27 | 110.53±  9.64 | 99.68±  13.64 | 91.91±  10.69 |
| GFAP Intensity  cortex |  | 2072.70±  252.23 | 2259.82±  220.15 | 2417.68±  110.16 | 2145.49±  223.73 |
| GFAP Intensity  dentate gyrus |  | 99.98±  11.67 | 110.78±  10.35 | 98.12±  9.77 | 90.08±  11.14 |
| GFAP Morphology  cortex |  | 41.19±  25.55 | 54.18±  17.67 | 49.37±  9.02 | 46.26±  9.87 |
| GFAP Morphology  dentate gyrus |  | 27.24±  4.54 | 50.44±  19.93 | 35.46±  5.62 | 34.88±  11.23 |
| Elevated Plus Maze |  | 73.26±  22.84 | 77.50±  21.58 | 86.65±  18.58 | 77.67±  18.89 |
| Novel Object Recognition |  | 0.36±  0.25 | -0.01±  0.17 | 0.32±  0.13 | 0.33±  0.18 |
| Y-maze |  | 0.35±  0.18 | 0.06±  0.05 | 0.36±  0.16 | 0.39±  0.17 |

Note. M and SD represent mean and standard deviation, respectively.


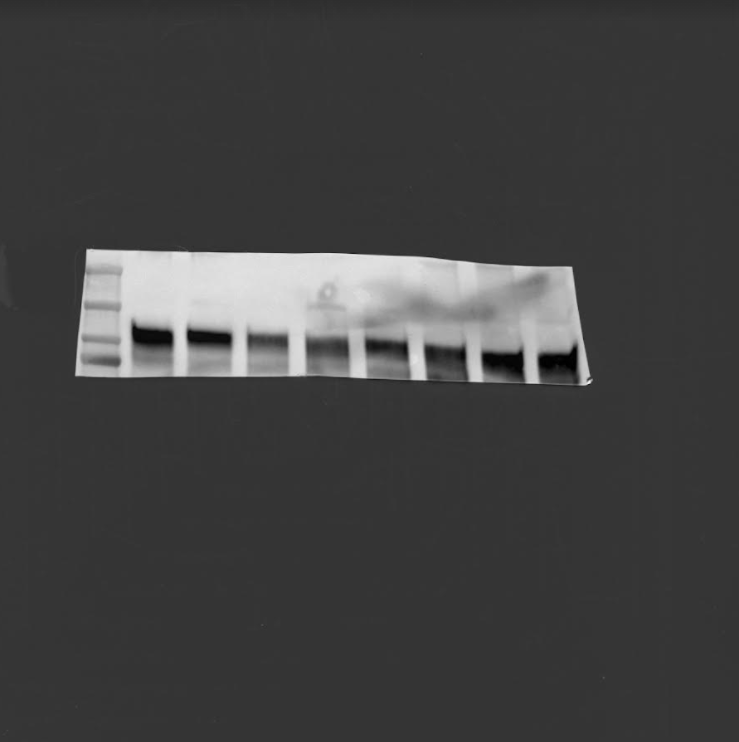


**Figure 1**. SIRT1 in the hippocampus. Image of gel electrophoresis followed by immunoblot analysis using antibody against SIRT1 in the hippocampus. The membrane was cropped immediately after the transfer stage due to the usage of two different antibodies on the same blot membrane.


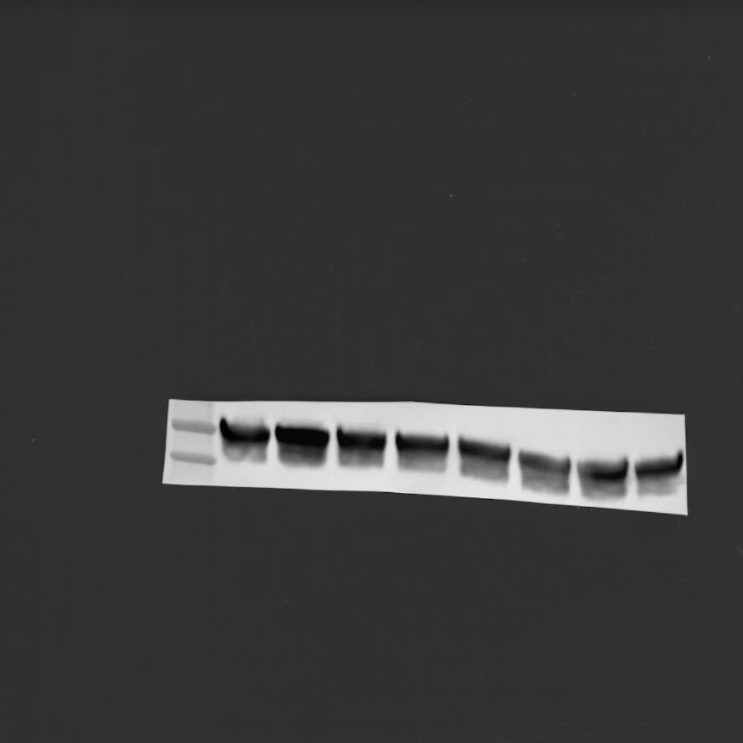


**Figure 2.** α-tubulin in the hippocampus. Image of gel electrophoresis followed by immunoblot analysis using antibody against α-tubulin in the hippocampus. The membrane was cropped immediately after the transfer stage due to the usage of two different antibodies on the same blot membrane.


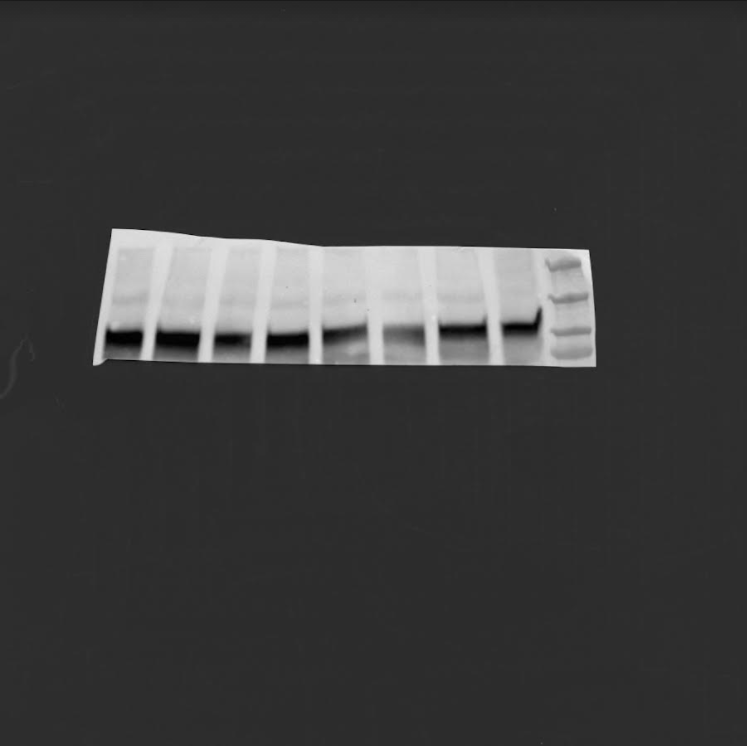


**Figure 3.** SIRT1 in the Cortex. Image of gel electrophoresis followed by immunoblot analysis using antibody against SIRT1 in the cortex. The membrane was cropped immediately after the transfer stage due to the usage of two different antibodies on the same blot membrane.


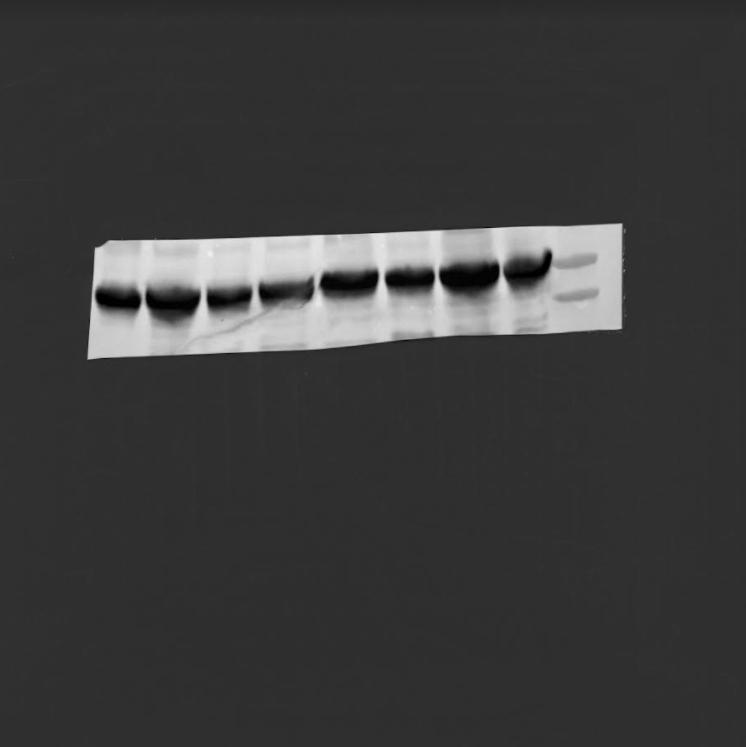


**Figure 4.** α-tubulin in the cortex. Image of gel electrophoresis followed by immunoblot analysis using antibody against α-tubulin in the cortex. The membrane was cropped immediately after the transfer stage due to the usage of two different antibodies on the same blot membrane.

**Table 3.**

Number of animals used for each analysis per group.

|  |  | **Controls** | **mTBI** | **KD** | **mTBI + KD** |
| --- | --- | --- | --- | --- | --- |
|  |  | **(Excluded/ Total)** | | | |
| Blood Ketone |  | 0/8 | 0/6 | 0/6 | 0/5 |
| SIRT1 cortex |  | 0/7 | 0/6 | 0/5 | 0/7 |
| SIRT 1 hippocampus |  | 0/7 | 0/6 | 0/5 | 0/7 |
| NeuN cortex |  | 0/5 | 0/5 | 0/3 | 0/4 |
| NeuN dentate gyrus |  | 0/5 | 0/5 | 0/5 | /15 ^a^ |
| Astrocyte I cortex |  | 0/5 | 0/5 | 0/5 | 0/5 |
| Astrocyte I dentate gyrus |  | 0/5 | 0/5 | 0/5 | 0/5 |
| Astrocyte M cortex |  | 1/5 ^a^ | 0/5 | 1/5 ^a^ | 0/5 |
| Astrocyte M dentate gyrus |  | 0/5 | 0/5 | 0/5 | 0/5 |
| Microglia cortex |  | 0/5 | 0/5 | 0/5 | 0/5 |
| Microglia dentate gyrus |  | 0/5 | 0/5 | 0/5 | 0/5 |
| Elevated Plus Maze | 7 days | 0/10 | 1/10 ^b^ | 1/14 ^b^ | 0/16 |
|  | 30 days | 0/10 | 0/10 | 0/10 | 0/10 |
| Novel Object Recognition | 7 days | 1/10^b^ | 2/10 ^b, c^ | 1/14 ^b^ | 1/16 ^b^ |
|  | 30 days | 0/10 | 0/10 | 0/10 | 2/10 ^b^ |
| Y-maze | 7 days | 0/10 | 1/10 ^b^ | 0/14 | 0/16 |
|  | 30 days | 0/10 | 0/10 | 0/10 | 0/10 |

^a^ Samples excluded due to technical problems.

^b^ Mice excluded from analysis for not reaching the 10% threshold duration of maze exploration.

^c^ Mice excluded from analysis due to health reasons.
